# Functional Identification and Characterization of Genes Cloned from Halophyte Seashore Paspalum Conferring Salinity and Cadmium Tolerance

**DOI:** 10.3389/fpls.2016.00102

**Published:** 2016-02-09

**Authors:** Yu Chen, Chuanming Chen, Zhiqun Tan, Jun Liu, Lili Zhuang, Zhimin Yang, Bingru Huang

**Affiliations:** ^1^Department of Turfgrass Science, College of Agro-Grassland Science, Nanjing Agricultural UniversityNanjing, China; ^2^Department of Plant Biology and Pathology, Rutgers, The State University of New JerseyNew Brunswick, NJ, USA

**Keywords:** seashore paspalum, yeast, cDNA library, salinity-tolerance, Cd-tolerance

## Abstract

Salinity-affected and heavy metal-contaminated soils limit the growth of glycophytic plants. Identifying genes responsible for superior tolerance to salinity and heavy metals in halophytes has great potential for use in developing salinity- and Cd-tolerant glycophytes. The objective of this study was to identify salinity- and Cd-tolerance related genes in seashore paspalum (*Paspalum vaginatum*), a halophytic perennial grass species, using yeast cDNA expression library screening method. Based on the Gateway-compatible vector system, a high-quality entry library was constructed, which contained 9.9 × 10^6^ clones with an average inserted fragment length of 1.48 kb representing a 100% full-length rate. The yeast expression libraries were screened in a salinity-sensitive and a Cd-sensitive yeast mutant. The screening yielded 32 salinity-tolerant clones harboring 18 salinity-tolerance genes and 20 Cd-tolerant clones, including five Cd-tolerance genes. qPCR analysis confirmed that most of the 18 salinity-tolerance and five Cd-tolerance genes were up-regulated at the transcript level in response to salinity or Cd stress in seashore paspalum. Functional analysis indicated that salinity-tolerance genes from seashore paspalum could be involved mainly in photosynthetic metabolism, antioxidant systems, protein modification, iron transport, vesicle traffic, and phospholipid biosynthesis. Cd-tolerance genes could be associated with regulating pathways that are involved in phytochelatin synthesis, HSFA4-related stress protection, CYP450 complex, and sugar metabolism. The 18 salinity-tolerance genes and five Cd-tolerance genes could be potentially used as candidate genes for genetic modification of glycophytic grass species to improve salinity and Cd tolerance and for further analysis of molecular mechanisms regulating salinity and Cd tolerance.

## Introduction

Fresh water availability for agricultural and horticultural irrigation has decreased, whereas there has been an increased use of recycled and effluent water for irrigation, which contains a high content of salinity and heavy metals that leads to soil salinization and heavy metal contamination (Uddin et al., 2011; Azevedo et al., 2012; Pessarakli, 2014). Salinity and heavy metals, such as cadmium (Cd), accumulation can be detrimental for plant growth and development (DalCorso et al., 2010; Chen et al., 2015a). Functional characterization of genes conferring plant tolerance to salinity or Cd will help to further understand the molecular mechanisms controlling abiotic stress tolerance. Global gene expression analysis of salt-tolerant *Arabidopsis thaliana* suggests that many salt-related genes are also associated with other abiotic and biotic stress responses (Chan et al., 2011). Furthermore, identification of salt- or Cd-related genes is important for developing plant germplasm with improved salinity or Cd tolerance in salinity-affected or Cd-contaminated areas.

Full-length cDNA Over-eXpressor (FOX) gene hunting system has been recently used in various plant species to detect and identify novel genes associated with stress tolerance through heterologous overexpression of full-length cDNA libraries in microorganisms or in the model plant *Arabidopsis* (Eswaran et al., 2010; Higuchi et al., 2011; Sakurai et al., 2011). Yeast has been utilized for functional assays or screening of plant stress-tolerance genes due to the simplicity as single cells, rapid growth, and the availability of mutants sensitive to various abiotic stresses (Qiu, 2012). Several Cd-tolerance related genes, including aquaporin and protease inhibitors and metallothionein, were identified from rice (*Oryza sativa*) with FOX-gene hunting system, as those genes could restore Cd tolerance in Cd-sensitive yeast mutants (Wang et al., 2012). Few studies reported that *phytochelatin synthase* gene (*PCS*) could be related to Cd-detoxification (Brunetti et al., 2011). However, the genes underlying Cd tolerance are largely unknown. Thirty-two full length salinity-tolerance related genes from physic nut (*Jatropha curcas*) and sixteen salinity-tolerance related genes from zoysiagrass (*Zoysia matrella*) were isolated with the yeast FOX-gene hunting system (Eswaran et al., 2010; Chen et al., 2015b). *OsMPG1* and *OsKAT1* were also identified in rice and confirmed for salinity tolerance (Obata et al., 2007; Kumar et al., 2012). Genes related to salinity tolerance are numerous (Gupta and Huang, 2014), and the most studied genes include those serving functions in salinity exclusion (i.e., *Salinity Overly Sensitive* 1, *SOS1*; Zhu et al., 2007; Yang et al., 2009), salinity compartmentalization (i.e., *vacuolar H*^+^*-pyrophosphatase, VP*; Chen et al., 2015a), and osmotic adjustment (i.e., *pyrroline-5-carboxylate synthetase, P5CS*; Hur et al., 2004). Salinity survival-related mechanisms, particularly for halophytic plant species that adapt to chronically severe salinity conditions, are yet to be completely understood and deserve further exploration.

Seashore paspalum (*Paspalum vaginatum*) is a halophytic perennial grass species which is known for its superb Cd and salinity tolerance, and it has been mainly used as a turfgrass in salinity-affected areas and for phytoremediation in Cd-contaminated areas (Chen et al., 2006; Wang, 2010; Liu et al., 2012; Uddin et al., 2012). Previous studies showed that seashore paspalum can grow well in soil solution with up to 48 dS m^−1^ of salinity and 254 mg Cd kg^−1^, whereas most glycophytic grass species cannot tolerate to such high levels of salinity or Cd (Flowers and Colmer, 2008; Wang, 2010; Uddin et al., 2012). Exploring the genes associated with both salinity and Cd tolerances in this unique perennial grass species will have a great potential for further understanding the molecular mechanisms involved in regulating salinity and Cd tolerances and for genetic modification of glycophytic grass species to improve salinity and Cd tolerances. The objective of this study was to identify salinity- and Cd-tolerance related genes in seashore paspalum using yeast cDNA expression library screening or FOX-gene hunting method. Stress-related genes are often induced by their respective stress conditions (Song et al., 2012; Chen et al., 2013, 2014). Therefore, qRT-PCR analysis was performed to detect the expression level of these candidate genes cloned from seashore paspalum under salinity- or Cd-stress conditions.

## Materials and methods

### Plant materials, growth conditions, and treatments

Plants were propagated from stolons of seashore paspalum “SeaIsle 2000” plants. The stolons were cut into 4–5 cm segments including two nodes of each segment and hydroponically cultured in plantstic containers (23 cm length × 23 width × 18 cm depth) filled with half-strength Hoagland's nutrient solution (Hoagland and Arnon, 1950). The nutrient solution was changed weekly to ensure adequate nutrition.

After roots and shoots were generated from the stolons, plants were transferred to half-strength Hoagland's nutrient solution containing 1 mM CdCl_2_ for 48 h to impose Cd treatment. For the salinity treatment, roots were exposed to lower salinity levels (100 and 200 mM NaCl for 2 h each) to avoid salinity shock and then to 250 mM NaCl for 48 h in the half-strength Hoagland's nutrient solution. After Cd or salinity treatment, the whole plant including leaves, stems, and roots (~9.5 g fresh weight, mixture combining NaCl and Cd-treated plants) was collected from both NaCl and Cd treatments, frozen in liquid nitrogen, and then stored in a freezer at –80°C for library construction. In addition, the ion contents (K, Na, Ca, Fe, and Cd) of leaves and roots after 24 h of Cd or salinity treatment were detected using an inductively coupled plasma mass spectrometer (ICP-MS), which exhibits significant increase of endogenous toxic elements (Na and Cd) and decrease of other essential elements (K, Fe, and Ca) under exogenous salinity or cadmium treatment (Table S1). Leaves and roots were separately sampled at 0, 1, 3, 6, 24, or 48 h of 250 mM salinity or 1 mM Cd treatment, immediately frozen in liquid nitrogen, and stored at –80°C for qRT-PCR analysis of candidate gene expression. Each treatment was replicated in three hydroponic containers representing three biological replicates and each replicate with multiple plants, providing sufficient tissue samples for analysis.

The experiment was conducted in a climate-controlled room (Jinan, Shandong, China) with 14 hd-1 photoperiod, photosynthetically active radiation of 850 μmol photons m^−2^ s^−1^, day/night temperature of 28/25°C and relative humidity of 60%.

### cDNA expression library construction and quality assays

The total RNA from roots, stems, and leaves of seashore paspalum was extracted using Trizol RNA Kit (Invitrogen), and mRNA was purified with Dynabeads® mRNA Purification Kit (Invitrogen). The integrity and purity of total RNA extracted from the plants were detected by 1% Agarose gel electrophoresis and Nucleic Acid Analyzer (Thermo nanodrop, USA), and purified mRNA was subjected to the same analysis.

The cDNA entry library was constructed using the SuperScript® Full Length cDNA Library Construction Kit II, including reverse transcription with primer Biotin-attB2-Oligo(dT), RNase I treatment and first-strand cDNA enrichment with Cap antibody, double-stranded cDNA synthesis and fractionation followed by attB1-adapter connection, BP reaction with cDNA and pDONR/Zeo vector, and ElectroMAX™ DH10B™ T1 transformation by Electroporation. Thousand-fold diluted library Bacilli were cultured overnight on solid medium (LB + 50 mg L^−1^ Zeocin) and the clones were counted. Twenty-four clones were selected for PCR reaction performed with the following pDONR/Zeo vector universal primer pair (F1/R1:TCCCAGTCACGACGTTGTAAAACGACGGCCAGTCTT/AGAGCTGCCAGGAAACAGCTATGACCATGTAATACGACTC). Sequencing was performed on 32 selected clones.

The mixed plasmids were isolated with PureLink® 96 Plasmid Purification System (Invitrogen), and an LR gateway reaction was performed with yeast expression vector pDEST52 (Invitrogen). The reaction products were transformed into ElectroMAX™ DH10B™ T1 competent cells, creating the expression library. A similar confirmation of the library was performed as above using the pDEST52 vector universal PCR primer pair F2/R2: TAATACGACTCACTATAGGG/ATCGAGACCGAGGAGAGG. The pDEST52 expression library plasmids were separated with PureLink® 96 Plasmid Purification System (Invitrogen), and then for yeast screening.

### Yeast transformation and gene mining

Cd-sensitive mutant yeast strain *ycf1* (his3Δ1; leu2Δ0; met15Δ0; ura3Δ0; YDR135c::kanMX4) was obtained from Euroscarf (Y04069, University of Frankfurt, Germany). PDEST52 expression library plasmids were transformed to *G19* salinity-sensitive mutant cell (*ena1-4* yeast mutant) or Cd-sensitive mutant *ycf1* using PEG-lithium acetate-based transformation protocols (Clontech). Transformed plasmids were then plated on SD solid medium with 500 mM NaCl or 100 μM CdCl_2_ lacking uracil and histidine, and placed at 30°C for 3–7 days until single clones appeared. Single clones that survived treatment with either 500 mM NaCl or 100 μM CdCl_2_ were selected and cultured overnight, and then streaked with different dilution production (10^2^, 10^3^, 10^4^, and 10^5^) on control as well as the salinity- or Cd-selection plates. Growth of transformed clones with PDEST52 expression library plasmids was compared with *G19* or *ycf1* cells transformed with the pDEST52 empty vector on the same salinity or Cd medium plates. The yeast plasmid of validated salinity-tolerant clones was extracted and transformed to TOP10 *E. coli* cells for sequencing (Invitrogen). Sequence analysis was performed following BLASTX (http://blast.ncbi.nlm.nih.gov/Blast.cgi).

### Expression vector reconstruction and stress-tolerance validation

To avoid the salinity- or Cd-tolerance achieved from co-transformation with multiple different genes in yeast, we reconstructed the ORF sequence of each screened gene into the yeast expression vector and retransformed to yeast cells to confirm their functionality. The full-length ORFs were PCR amplified with primer pairs including the enzyme-digestion connector and then digested for further connecting to the same digested entry vector pENTR1A. The pENTR1A constructs were then subjected to LR recombination reactions with pDEST52 expression vectors. The constructs were retransformed into yeast and then salinity- and Cd-tolerance were verified using the above method. The transformed and non-transformed yeast cells were cultured in Petri dishes containing 300 or 500 mM NaCl for salinity treatment and 100 μM Cd for Cd treatment.

### qRT-PCR analysis of expression levels of candidate salinity- or Cd-tolerance genes under salinity or cadmium stress

The samples of leaves or roots sampled at 0, 1, 3, 6, 24, or 48 h of 250 mM salinity or 1 mM Cd treatment were used separately for RNA extraction using the RNApure reagent (Yuanpinghao Biology, Tianjing), and cDNA first strand was produced using PrimeScript RT reagent Kit with gDNA Eraser (TaKaRa, Dalian) following the manufacturer's instructions. A 10-fold dilution of cDNA was used for qRT-PCR reaction as template. Primer pairs of 18 candidate salinity-tolerance genes, five candidate Cd-tolerance genes, and a reference gene Elongation factor-1α (*SpEF1*α, GenBank accession number: KU049721) were produced following Primer Premier 5.0 software, as a principle of primer length 18–25 bp, GC content 40–60%, melting temperature 55–65°C, and amplicon lengths 80–300 bp. qRT-PCR detection was performed using a LightCycler 480 II instrument (Roche, Switzerland), and a reaction of a 15 μL mixture containing 7.5 μL 2X SYBR I master (Roche, Switzerland), 5 μL of diluted cDNA, 0.4 μL each primer (10 μM), and 1.7 μL ddH_2_O. The reaction procedure included an initial 10-min denaturation of 95°C, 40 cycles amplification (95°C for 15 s, 58°C for 15 s, and 72°C for 30 s), and then melting curves were produced at 60–95°C. Gene relative expression level was calculated following the 2^−ΔΔCt^ method. Three biological replicates were used for significance detection by one-way analysis of variance (ANOVA) using a statistical program (SAS9.0, Cary, NC).

## Results

### Seashore paspalum cDNA library construction

**Figure 1:**
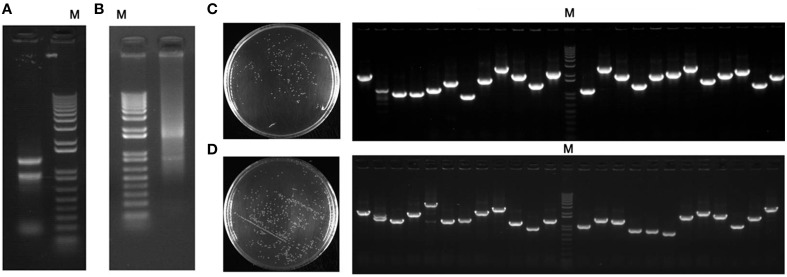
**Expression library procedure of seashore paspalum**. **(A)** Analysis and detection of total RNA extracted from roots, stems, and leaves of seashore paspalum by 1% Agarose gel electrophoresis; **(B)** Analysis and detection of mRNA purified from total RNA extracted from roots, stems, and leaves of seashore paspalum by 1% Agarose gel electrophoresis and spectrophotometer (NanoDrop2000, Thermo Scientific, Massachusetts, USA); **(C)** titer and inserted fragment detection of entry cDNA library; **(D)** titer and inserted fragment detection of expression cDNA library. M represents invitrogen 1 kb plus ladder.

RNA concentration and the quantity extracted from the whole plant of seashore paspalum (leaves, roots, and stems) were 905 ng μL^−1^ and 543 μg, respectively, and the A260/A280 value was 2.03 (Figure 1A). The mRNA exhibited excellent quality (Figure 1B) with a A260/A280 ratio of 2.19, and total mRNA concentration and quantity was ~76.5 ng μL^−1^ and 22 μg, respectively.

The total library included 9.9 × 10^6^ clones, which represent most of the genes expressed in seashore paspalum (Figure 1C). The inserted fragment length of 24 selected clones ranged from 0.8 to 2.5 kb with an average size of 1.48 kb. Thirty-two selected clones were sequenced for detection of full-length rate (Table 1). BLASTX analysis showed that ATG start codon was found in all 32 sequences (NCBI accession number: KT203458–KT203489), and 5′UTR length ranged from 10 to 580 bp, with the average size of 105 bp (Table 1). This data demonstrated that full-length ratio of cDNA entry library was 100% and could be further used for the construction of expression library. The analysis of the pDEST52 expression library showed that the library capacity was 2 × 10^7^ and average recombination size was 1.54 kb (Figure 1D).

**Table 1:** **5′UTR sequence analysis of 32 randomly selected clones in entry library of seashore paspalum**.

**No**.	**Accession number**	**Prediction protein**	**Upstream sequence of Start codon ATG**
1	KT203458	Aspartic proteinase oryzasin-1-like [*Setaria italica*]	ACGGTTTCTGCCTTTGTCTGATCGCAACC**ATG**GGA
2	KT203459	S-Adenosylmethionine decarboxylase [*Sorghum bicolor*]	GCTTCTTGTTGCCGAAGAATTTCCTCCCCCTC GCCGCCGCCGCCGCCGCTCGTCTCGCTGAGAG AAGCTAACTGGGCCGGGGAACTCGCTTGTCTC TAGGGAGAAGAGGATCCTTCCCGCTGGTCTCA GCCCGGGGTCTGCTGCGTCGGAAGGCGGTGAA GGGGGGAGATTCGTGAGATCGTTTCCGGATCA CGCGTGCGCGCTCGGGAATTTCGGGGGTATCC ACACATAGCTTCGTCGAATTGAAATTGATGTA CTAATGGAGTCCAAGGGTGGCAAGAAGTCTAG CAGTAGTCGTTCCATGATGTATGAAGCTCCCC TTGGCTACAGCATTGAGGACGTTCGACCTGCT GGAGGCGTGAAGAAGTTCCAGTCTGCTGCTTA CTCCAACTGCGCGAAGAAGCCATCCTGATATC CCTTTGGGCTTCCCTTTCCTAGTAGTATAGGA TTTCTTTTCTAACGCTTTGATTCTGACCAATC TCTCTGGCCTGCTGCTTCCTGATAATCAACCA GTTCCCCAGTCTTGCTCTCTGCACTCCTCCCT CCATCTCTGGCATCGTGTGCCGATTCACCTGC TTCA **ATG**GCT
3	KT203460	Homeobox-leucine zipper protein HOX1-like [*Setaria italica*]	CCTCCTCATTGCCCACGCCGAGACCGTTTCTT TCTCTGCAACCAGCGGCGGGCTCCGGTCTGGA GTAATCGTCGTCAGTGGAG**ATG**ATG
4	KT203461	Palmitoyl-protein thioesterase 1-like [*Setaria italica*]	GTCCCGACCCGAGAGCGAGAGGAAGAGAAGAG GGGCTGCTGCCTGCTGGATCGAGTTGTTGGAG TGGAATCGGACCCCGGCCGCCGGAATCCGACC CCG**ATG**CGG
5	KT203462	60S Ribosomal protein L14-1-like isoform X2 [*Setaria italica*]	CTCGCCGTCTCGCTCGCAGCCGCTTGCCGCCG CCGCCGCTCGCCTCTTCGACCGCCACGAGG**ATG**CCG
6	KT203463	Hypothetical protein OsI_03805 [*Oryza sativa Indica* Group]	CAACAACGGCCAGCTGAGCTGAGCCGAGCCGA GCCGAGCGCACGCAGCTCGTTCGTCCTCGTCC TCGATCGATCGGCCTAGCTATTAGCTCCTTCC TGCCGCCATACCATTACCATATGAGGTAGCGC AGGCGCAGGAGCCGCCGGCGGGGCTGGAAGGT GAGGTGGAGAGAGATATAGAGAAGGATCGGCC GGCCGGTACGGTAGAGGTGCGTGGTCTGGTCA GGCTCCGGGCGGGATCGATCATATCGAG**ATG**GAG
7	KT203464	ABC-type Co^2+^ transport system, permease component [*Zea mays*]	GCGCTCGTCGCCGTCGTCTTGAGCCGTGAGAC GACACCAAGATAAAC**ATG**GCC
8	KT203465	Glutathione transferase [*Zea mays*]	ACAGCAAGCATCCTGCACGTTCGCAAGCTCTC TCTCGCACAGGGCACAGAGGAGGAAGGAAGGA GATCGAGGTCGAGGCC**ATG**GCG
9	KT203466	40S ribosomal protein S7-like [*Setaria italica*]	CTCCTCCTCGAGCGCAGGGTCCGGCGGCGAAG GGAAGGCAAG**ATG**TAC
10	KT203467	Nicotianamine synthase1 [*Zea mays*]	GTTGTCGAGCACTTGCCACTCTTGATCGAGCT AAAGCCTAAAGACATCTCATCCGCTGCGTCGT CGTCCCTAGCTCATCTTCCCAAGTCCAACCGT AGAAAGTACTACAGCTGCC**ATG**GAG
11	KT203468	Transcription factor bHLH96-like [*Setaria italica*]	ACTGCATCCACGCCGGCCGGCCGGTGATCGAG CCGCCGCTCTAGTGCTGCTTACTACTCTCTCT CGTTCCCGCCGCGTCCTCCTCGAGTAGGCCGG TGATCGATTCTCGCGTGCCGCC**ATG**GCG
12	KT203469	Remorin [*Zea mays*]	CAGCAACAGCTAAGCTTCGCCCACCAGACAGC AACAGCATC**ATG**GCT
13	KT203470	Uncharacterized protein LOC101767282 [*Setaria italica*]	ATCAGCTGGAAATCGATCGATTCTTACCAAAA TCCAGCATCTACACACCTCACAACTTCACAAG ATCCTCCTTTCTTCTTCTTCTTCTTCTTCCTT CCCTGCTGCTGCTGCTGCTGCTGGTCACCAGT CACCACCCTCATCATTTCTTCCCCGGCAGCCT ATAACCTTCCTGCCTGCCTTCCCTGCCAGGCC ACCCAAGCCTCCAAGAGATAGATCATATATTG **ATG**GAT
14	KT203471	Serine/Threonine protein phosphatase superfamily protein [*Zea mays*]	CCCCCTCCCTCCTTCCCACCTCTCGTTCTCTC CCCCTCTTCTCCACTTCCGTCCTCACGCGCGC GCGCGCGTCCAGATCTAGGGTTCCATCCGCGG CCAAG**ATG**AGC
15	KT203472	Chaperone protein dnaJ 8 [*Setaria italica*]	AAACCAAAGGCACCTCTCCAGTTCTCCACTTC TCCTCCCGTAGCGCGCGCTCTCTCGGACACAC AAGTCGCTTGCTTTGCTTCGCGCTCCTGGTGC TCGTTGCTTTCGATCCTCTCCGGCCGGTGATC TCGCTCGCTCGCGGCGGTGCTAGCTGTGGAA**ATG**GCC
16	KT203473	Probable sodium/metabolite cotransporter BASS2, chloroplastic-like [*Setaria italica*]	CCGGCGCTTTCCACGGGCTTCGGAAGCCACAA CTTTCCGCATCTCGTGCTTTGCCTCCTAGGTT CCTCCGC**ATG**GCG
17	KT203474	GTP-binding nuclear protein Ran-A1 [*Zea mays*]	AATTCTAGGGTTTTGCTGCTCCCTGTGCTCTG AAGCCCGCATCGCCGCATCCGGCGAG CTCCTCTCGGGCGACCGAA**ATG**GCG
18	KT203475	30S Ribosomal protein S10, chloroplastic-like isoform X2 [*Setaria italica*]	ACGCGGCAAACGCCACTGCTCCTCTCCTCATC CTCTTCCTCTTCCCCGCCTCCCTCGCC TCGCC**ATG**GCC
19	KT203476	Uncharacterized protein LOC101762337 [*Setaria italica*]	ACATCACATACATGCATGTAGGCTCACAAAAA CCCTAGTCGCAGCACCTTGCTGGC**ATG**GCT
20	KT203477	Phosphoenolpyruvate carboxylase 1 [*Aegilops tauschii*]	GCCTCGCTCAGCAGCAAAAACACGCGGCCCTT GCTCTTGCTTCGTTCTCGCTTCCCGCCCCGCC **ATG**GCG
21	KT203478	Chlorophyll a-b binding protein M9, chloroplastic-like [*Setaria italica*]	CTCTTCTGCAGAGTATAGTGTAACAGTAGACC AGCAGTGCA**ATG**GCG
22	KT203479	Catalytic/Hydrolase [*Zea mays*]	CTCCTCTCTCGCATCTGGTTCGGAGAGCTCTC CTGAAGCCTCGAGACTCGTCTCGCCAGCCGCC TGCTCCGCCTCCGCCCCGGCTCTGCCGCTGCC TGCGCTAGCTCTCGCATTGCGCCCCGCGAAGG CCTGAGCTGCTAGCTAGCTCCTGCTCCTTCTT CACCCCGCACACACAGTTCCGCGCGCC**ATG**GAG
23	KT203480	Nuclear cap-binding protein subunit 2-like isoform X4 [*Setaria italica*]	CACACCCACA**ATG**GCG
24	KT203481	Copper transporter [*Triticum aestivum*]	GCTCCCCACCACCACCAAGCACCTGCCTTTAC CCCTCTCCCCCCTCCCAAGAGGCCAGGAGGAC GAGGAGCCCGGCGCCGGCGTGCCAGCCCTGCC AGTGCCGCGCGGATCCGCCGCCGGCGCGCGGC CGTCGCTCTCCCGGCTCGACGCAGCAGGTGAC GCCAAC**ATG**GCG
25	KT203482	Cytochrome P450 CYP78A53 [*Zea mays*]	ATCTGATCAAACAATCGAACCCGTGACCACCG CTCCTTGCTTCTCTGCTGGTGGTCTTAACTCT TCTTCTTCTTCCCACTCGTCAGTGCCATTCCA CACGACCGGACCACCAT**ATG**GCG
26	KT203483	DRE-binding protein 3 [*Zea mays*]	CAAGCACAACTCAAGCAGCAGCAGCAGCAGAC AGCCACTCAGCTAGGCTAAAGCAATCGTTCCC CAGGGCGATTCAAGAACGAACAAG**ATG**TGT
27	KT203484	Non-specific lipid-transfer protein 2-like [*Setaria italica*]	GGGCATCCTCGATCGATCAGTTCCTCACTAGC TGCTAGTACTCATCATCACTCGCCCGCG**ATG**ACG
28	KT203485	Tonoplast dicarboxylate transporter-like [*Setaria italica*]	CCCGCAGCGCTGAGCGCAGCGACGGTTCTGTT GCTCCGGTTTCCTCGGCCTCGGCC**ATG**GAC
29	KT203486	Peroxidase 5-like [*Setaria italica*]	GCGAGTAGCGACACGGGGTCTCCACTTTGCGA TTGCAACTGCAGCCGCGCGCCGGATTAGCGGC TAAGCAAGCCATCCCAGGGGACAGAGCTCCAG GCACTCAAGCAGCCAGCCACAGCCCACACAGC CAGGGAT**ATG**GAA
30	KT203487	Malate dehydrogenase, cytoplasmic [*Zea mays*]	ATTTGGTCGACGCCTCCAAAGCCTCTCCCAAA CCTCCGCTTCCAGAACCTCCTCGAAGCTCCCC GCCACAGCCTCCACCCGCTCCG**ATG**GCG
31	KT203488	Putative signal peptide peptidase family protein [*Zea mays*]	CGCGGCTCTGCGCCATTCTCGAAGGTTCAACG GGCA**ATG**GCG
32	KT203489	Cysteine proteinase 1-like [*Setaria italica*]	CCGGAAAAAAAATCTAGCTCGACTCTGTCACC TCAAC**ATG**GCT

### Yeast transformation and clone selection

**Figure 2:**
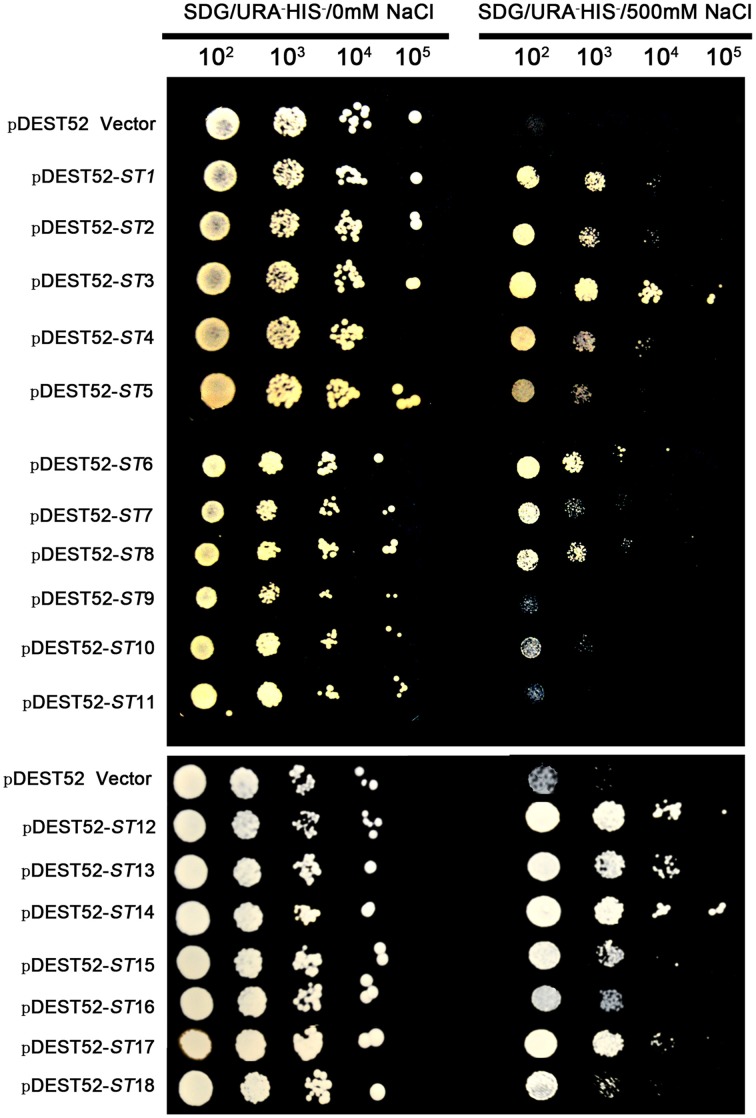
**Salinity-tolerance confirmation of 18 clones via library screening of seashore paspalum**. The overnight culture yeast were, respectively, diluted 10^2^, 10^3^, 10^4^, and 10^5^, and then the 5 μl dilution yeast grow on SDG with or without NaCl plates for 5 days.

**Figure 3:**
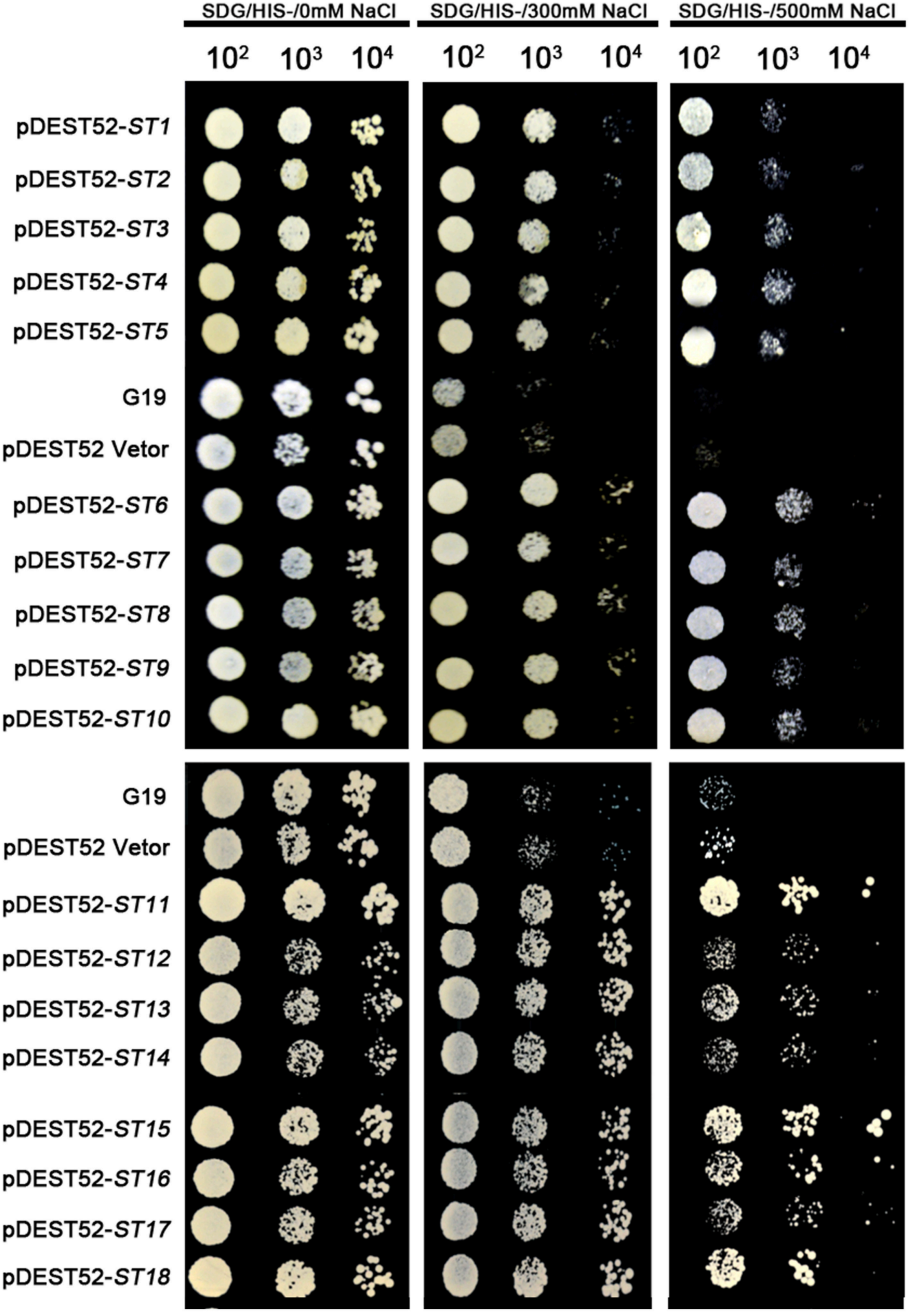
**Salinity-tolerance confirmation of retransformed yeast with 18 single genes of seashore paspalum**. The overnight culture yeast were, respectively, diluted 10^2^, 10^3^, 10^4^, and 10^5^, and then the 5 μl dilution yeast grow on SDG with or without NaCl plates for 5 days.

**Figure 4:**
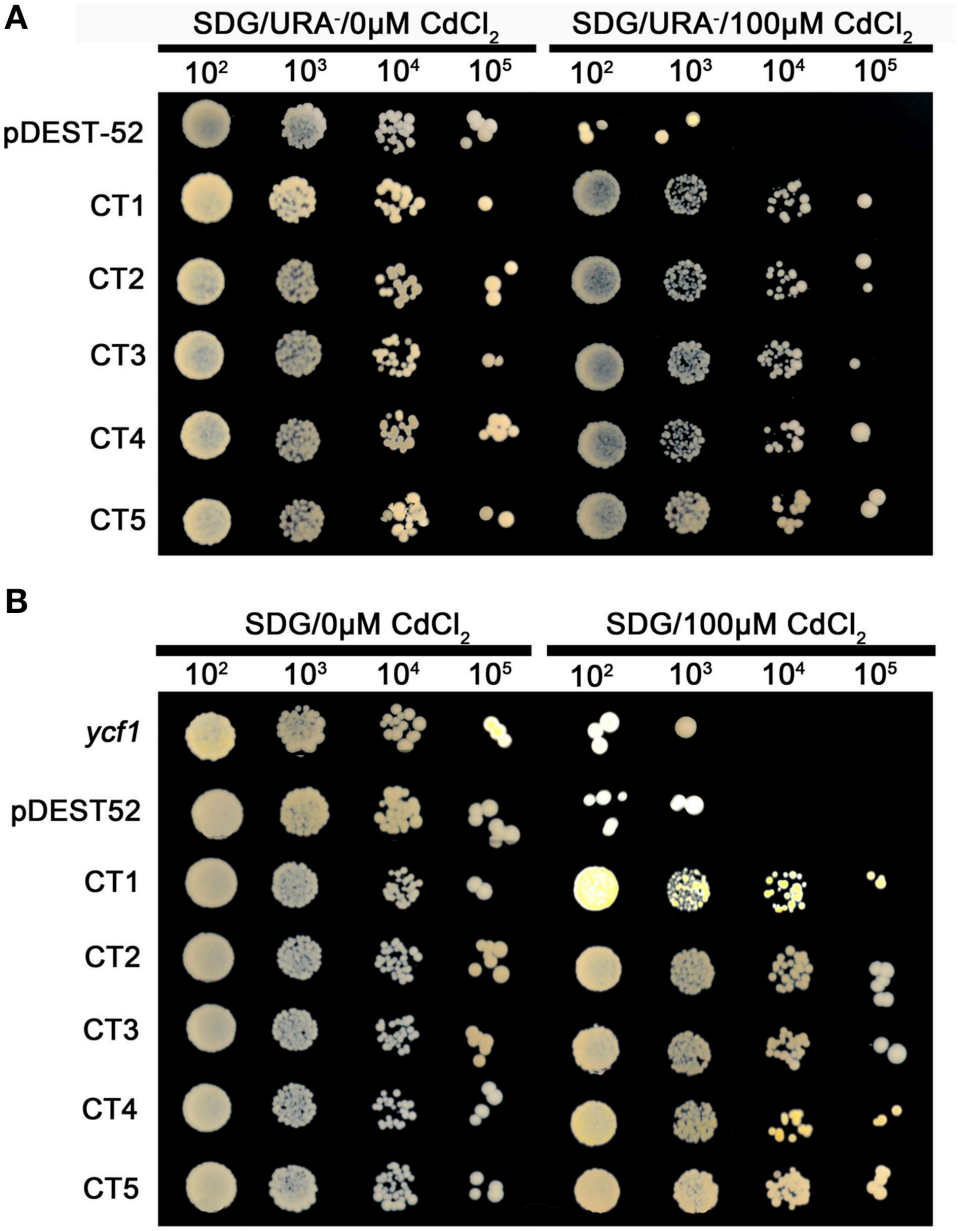
**(A)** Cd-tolerance confirmation of five clones via library screening of seashore paspalum; **(B)** Cd-tolerance confirmation of retransformed yeast with five single genes of seashore paspalum. The overnight culture yeast were, respectively, diluted 10^2^, 10^3^, 10^4^ and 10^5^, and then the 5 μl dilution yeast grow on SDG with or without Cd plates for 5 days.

The pDEST52 expression library plasmids were transformed into salinity-sensitive *G19* or Cd-sensitive *ycf1-*competent yeast cells. Among yeast cells transformed with pDEST52 expression library plasmids, 32 clones survived 250 mM NaCl treatment and 20 survived 1 mM Cd treatment. Through sequencing of these clones, 18 candidate salinity-tolerance genes and five candidate Cd-tolerance genes were identified. The yeast transformed with one of the 18 salinity-tolerance genes or the five Cd-tolerance genes exhibited more rapid growth in the culture medium treated with NaCl or Cd compared to the yeast transformed with the pDEST52 empty vector (Figures 2, 3, 4A). Yeast growth did not show differences between those transformed with the candidate genes from seashore paspalum and those with the pDEST52 empty vector under non-stress conditions.

### Gene sequence and functional analysis

The results of BLASTX analysis of cDNA sequences in the salinity- or Cd-tolerant clones were shown in Table 2 (NCBI accession number: KT203435–KT203457). The proteins encoded by 18 genes from the salinity-tolerant clones were found to be involved in iron transporter (ST2, ST12), photosynthetic metabolism (ST3, 4, 5), protein modification enzymes (ST6, 15), antioxidant metabolism (ST8, 9, 10), heat shock protein (ST7), vesicle-associated protein (ST11), Nop14-like family protein (ST13), choline-phosphate cytidylyltransferase (ST14), IQ-DOMAIN 14-like protein (ST16), metacaspase-5-like protein (ST17), BTB/POZ domain-containing protein (ST18), and a function-unknown protein (ST1). Five genes from Cd-tolerant clones were identified as phytochelatins synthase (CT1, 2), cytochrome P450 (CT3), a heat shock transcription factor (CT4), and UDP-glucose pyrophosphorylase (CT5).

**Table 2:** **Sequence analysis and function prediction of 18 salinity-tolerant and five Cd-tolerant candidate genes screened from cDNA expression library of seashore paspalum**.

	**Accession number**	**cDNA size (bp/aa)**	**Predicted function**
**SALINITY-TOLERANT CLONES**			
ST1	KT203435	961/227	Uncharacterized protein (UP)
ST2	KT203436	1599/370	Iron-regulated transporter (IRT)
ST3	KT203437	686/138	Photosystem I reaction center subunit psaK (PSAK)
ST4	KT203438	1105/268	Chlorophyll a/b-binding protein (LHCB)
ST5	KT203439	887/172	Early light-induced protein (Elip)
ST6	KT203440	1099/263	14-3-3-Like protein (14-3-3)
ST7	KT203441	948/153	Class 1 HSP (HSP)
ST8	KT203442	1552/378	Cysteine synthase (CS)
ST9	KT203443	1370/310	Aldo-ketoreductase (AKR)
ST10	KT203444	1270/246	L-Ascorbate peroxidase 2 (APX)
ST11	KT203445	1199/225	Vesicle-associated protein33 family (VAP33)
ST12	KT203446	1390/342	Iron-phytosiderophore transporter yellow stripe 1 (YS)
ST13	KT203447	2214/501	Nop14-like family protein (NOP14)
ST14	KT203448	1327/293	Choline-phosphate cytidylyltransferase B (CCT)
ST15	KT203449	2527/655	Leucine-rich repeat receptor-like protein kinase (LRR)
ST16	KT203450	2094/559	Protein IQ-DOMAIN 14-like (IQ14)
ST17	KT203451	1551/415	Metacaspase-5-like (MCP)
ST18	KT203452	1379/336	BTB/POZ domain-containing protein (BTB)
**CADMIUM-TOLERANT CLONES**			
CT1	KT203453	2080/499	Phytochelatins synthase (PCS1)
CT2	KT203454	1858/507	Phytochelatins synthase (PCS2)
CT3	KT203455	1808/534	Cytochrome P450 (CYP450)
CT4	KT203456	1647/435	HSFA4a
CT5	KT203457	1785/474	UDP-glucose pyrophosphorylase (UGP)

### Confirmation and qRT-PCR analysis of candidate genes for salinity and Cd tolerance

Different plasmids in the library carrying different genes could be incorporated into a single yeast cell. To avoid that possibility, we reconstructed the single gene vector (the ORF primer shown in Table 3) and separately transformed them into yeast cells for an independent test of each individual gene (Figures 3, 4B). All transformed yeast cells exhibited better growth than untransformed yeast with empty vectors under 300 or 500 mM NaCl, or 100 μM Cd (Figures 3, 4B).

**Table 3:** **Primer pairs of 18 salinity-tolerant and five Cd-tolerant candidate genes for PCR amplification of full-length ORF region from seashore paspalum**.

**Gene**	**Primer sequences 5′–3′ (ORF-F/ORF-R)**
ST1/*UP*	GTCGACATGCTCCTGAGGAGCAAGCCT/ GATATCTCGAGGACAGCTCCTGTGCCT
ST2/IRT	GGATCCGGATGTCGTCTTCGCAGGCA/ GATATCTCGCCCACTTGGCCATGAC
ST3/PSAK	GTCGACATGGCTTCCCAGCTCTCCGCCG/ GATATCTGCCGATGATCTGGTCGAGCG
ST4/LHCB	GTCGACATGGCGGCTCAGGCTCTCCTCT/ GATATCTGTGGAACTTGAGGCTGGTGA
ST5/Elip	GTCGACATGGCAGCCACGGTGATGG/ GATATCTCACGTTGACGAATGGCGCG
ST6/14-3-3	GAATTCGCATGGCGGGGCAGCAGA/ GATATCTCTGCTCATCCTCAGGCTT
ST7/HSP	GTCGACATGTCGCTCGTGAGGCGCAGCA/ GATATCTGCCAGAGATTTCAATCGCCTT
ST8/CS	GTCGACATGGAGAGGATGCTGACGAGGT/ GCGGCCGCGAGTCCACTGGCACTGGTTCCA
ST9/AKR	GTCGACATGGCGAGGCACTTCATGCTCAA/ GATATCTAAGTTCGCCATCCCAGAGGTCCT
ST10/APX	GGATCCGGATGGCGAAGGCCTACCC/ GCGGCCGCGACCCCAATTCAGAAAGTT
ST11/VAP33	GTCGACATGACCACCGCCGACTCC/ GATATCTTCTCTTCATGAAGAACCCT
ST12/YS1	GGATCCGGATGGGAGACGGTATGTACCA/ GCGGCCGCGAGGTTCCAGGTGTGAACTT
ST13/NOP14	GTCGACATGTCTGCTAGGGACTGGGA/ GATATCTCCGCCTCCTTTTTCTGCCCT
ST14/CCT	GTCGACATGGCGCGCGTGTCCAATGCCA/ GATATCTGGCTGCCACCACTTCCTGCA
ST15/LRR	GTCGACATGGGCGGCGCGGGCGCCG/ GATATCTCGCGGAGTGGGAGCTGGGGA
ST16/IQ14	GGATCCGGATGGGTAAGAAGGGAAACT/ GCGGCCGCGACTTGAAGGGCCTGCCGAG
ST17/MCP	GGATCCGGATGGGGGCGAAGCGCGCGGT/ GCGGCCGCGAGCATATGAAAGCCACATCG
ST18/BTB	GGATCCGGATGAACAGCGGTGGCGGCGG/ GCGGCCGCGATATGGGCTTCCAGACACCGA
CT1/PCS1	GTCGACATGGCGGCGGCGGCGCCGT/ GATATCTAGGGAATGGTGGCACAAGAT
CT2/PCS2	GTCGACATGGCGGCGGCCGTGGCGT/ GATATCTGCATTGCTGCTTAGATGATG
CT3/CYP450	GTCGACATGATCGTTCTTGGAGAAGC/ GATATCTTATAGCCCTGAGCTTGATC
CT4/HSFA4a	GGATCCGGATGGAGGCGGGCGGCGGG/ GCGGCCGCGAGGTTTTCTCTGCCGAGGT
CT5/UGP	GGATCCGGATGGCCGCTGCCGCCGCCG/ GCGGCCGCGAAAGATCCTCAGGGCCATT

**Figure 5:**
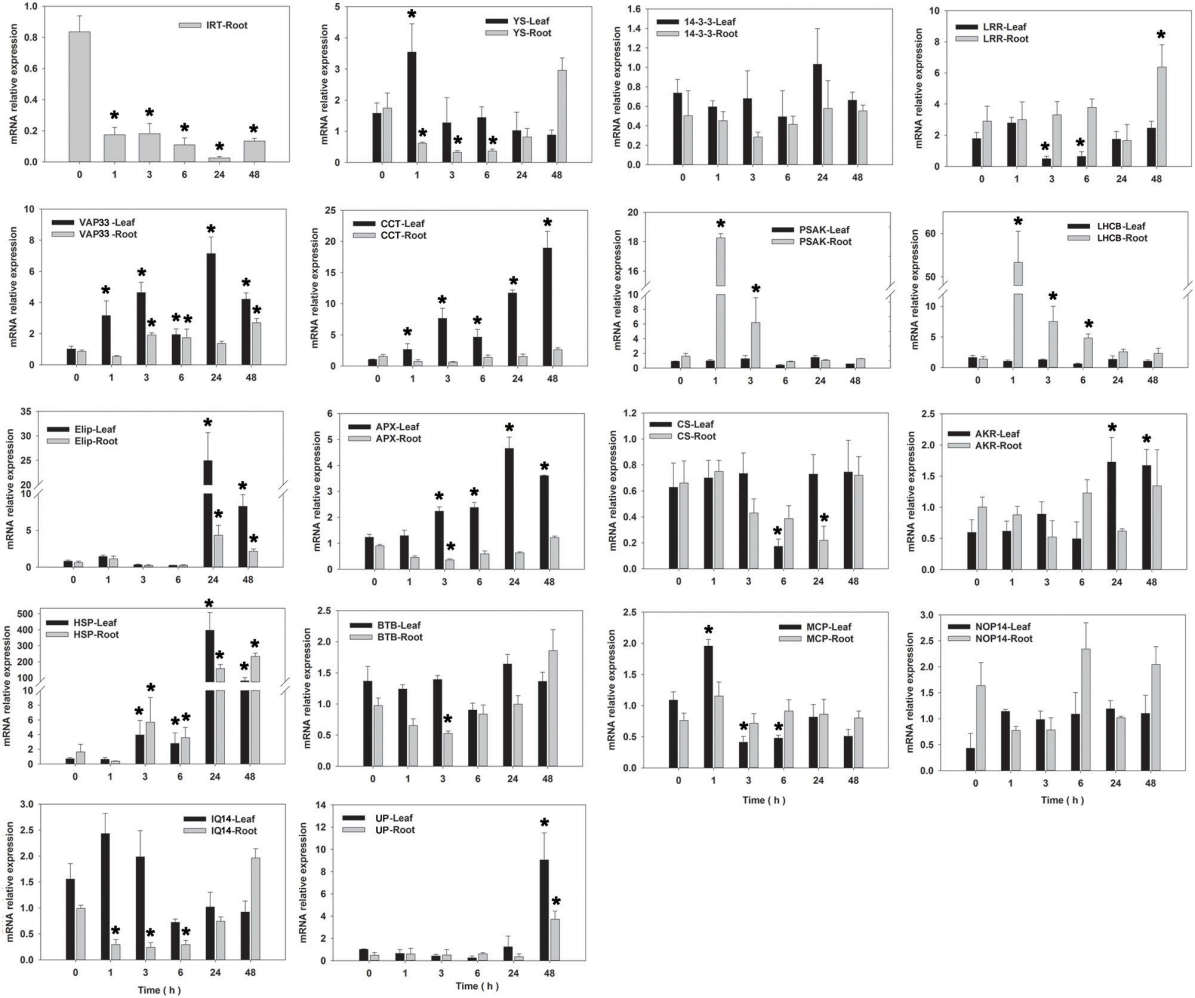
**qRT-PCR analysis of 18 salinity-tolerant candidate genes in roots and leaves of seashore paspalum under salinity stress at 0, 1, 3, 6, 24 and 48 h**. ^*^Represent significant difference (*p* < 0.05) under salt stress comparing to CK (0 h).

**Figure 6:**
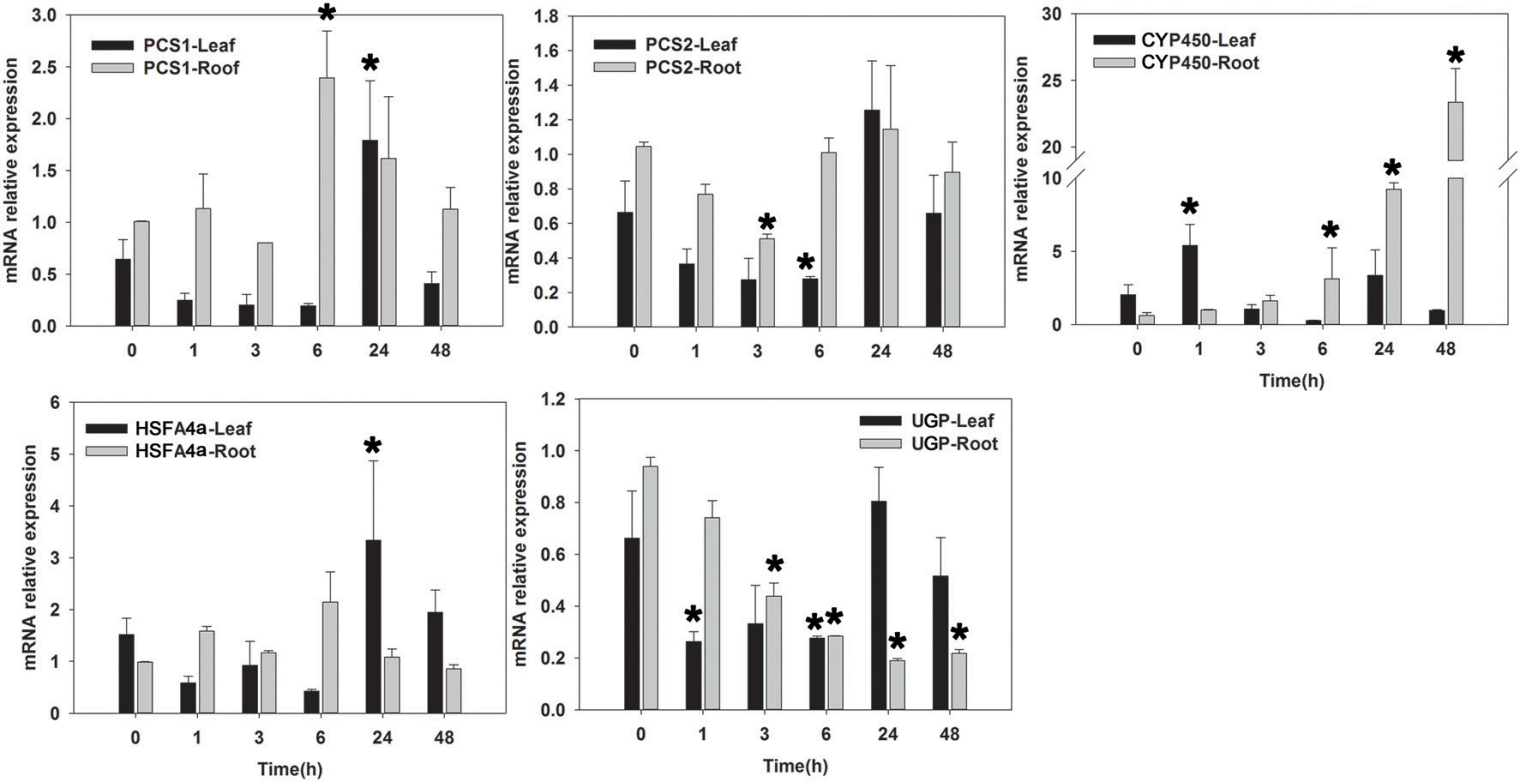
**qRT-PCR analysis of five Cd-tolerant candidate genes in roots and leaves of seashore paspalum under Cd stress at 0, 1, 3, 6, 24, and 48 h**. ^*^Represent significant difference (*p* < 0.05) under cadmium stress comparing to CK (0 h).

qRT–PCR analysis (primer shown in Table 4) was performed for determination of the expression level of 18 candidate salinity-tolerance genes under salinity stress conditions (Figure 5) or five candidate Cd-tolerance genes under Cd treatment (Figure 6) in two tissues (leaves and roots) of seashore paspalum. Under salinity stress, *IRT* was not detected in leaves, whereas its expression level decreased in roots. *YS* expression level did not change significantly in leaves during salinity treatment but decreased in roots within 24 h of salinity stress and then increased after 48 h compared to the non-stress conditions. *14-3-3* and *NOP14* transcript levels in leaves and roots were not altered by salinity stress. The expression of *AKR* in leaves and *LRR* in roots was induced at 24 h of salinity stress. *LHCB* and *PSAK* of roots and *MCP* of leaves exhibited up-regulation of expression at 1 h and thereafter down-regulated by salinity stress. *CS* expression in leaves or roots was declined under salinity stress at 6 or 24 h and then returned to the untreated level. Salinity stress significantly promoted the expression of *HSP, VAP33, Elip*, and *UP* in leaves and roots, and *APX* and *CCT* in leaves. The expression of *BTB* in roots at 3 h, *IQ14* in leaves and roots at 6 h, and *LRR* in leaves at 3 h were all reduced under salinity stress.

**Table 4:** **qRT-PCR primer pairs of 23 candidate tolerance genes in seashore paspalum**.

**Gene**	**Primer sequences 5′–3′ (RT-F/RT-R)**	**Amplicon length (bp)**
ST1/*UP*	CTCCCAGGAAGAAAGGCTG/GACGGTGATGAGGACAAGC	185
ST2/IRT1	CTCCAGCTCCTCTCCTTCCT/CCGTCGGTTCTCCGTTTT	100
ST3/PSAK	ACGACGCTGATGCTGTTCG/ACCTGCTTGGCCCTTGATG	297
ST4/LHCB	GTCAACAACAACGTGCTCACC/GCATCTCGATCGCTTCCA	141
ST5/Elip	CTGGAACGGACGATTCG/CGCAGACGCAGTGTTTTTGA	156
ST6/14-3-3	TGGTGGCGATGAAATGAA/AAGGCTACCCCAAGGGTTA	102
ST7/HSP	AGGACAAGAACGACAAGTGG/TGGACCAAGGACCATCAGTA	256
ST8/CS	GGGAGCGATACTTGTCTTCTG/CGGTGCCCTGTTTAGTTCTC	104
ST9/AKR	CCGCAGAGCGTTTACAAGA/CCACGATCCAATATGACAGG	245
ST10/APX	TTCCGCCCGCTTGTT/ATCCCCCCCCTTCTTTAG	237
ST11/VAP33	CGAGACATCAGCAGGCAA/GCACACAGAGAACACGTACAATC	171
ST12/YS	GAGGCTCGTGCCACTACCAA/TGACTCCTTGACTGTGTCACACAT	298
ST13/NOP14	CAAGGGAAAGGGCAGAAAAA/AGCAGCTCGCATTAAGGACA	170
ST14/CCT	GAGCAAAGGAATAAGCACTGG/CGATCATAACGGATACCCAAA	287
ST15/LRR	ATTACTGCTGCTGCTAGGTTCT/GTGTATTTGGCCTCCTTTTTCT	194
ST16/IQ14	TGTGGTTTGGTGTGGGTTTC/CGCATGTACGGTTGTTCGAT	159
ST17/MCP	TGAATGCCTGAATCACTGGG/AGCAAAAAGCGTAACCACC	125
ST18/BTB	ACCTACCTGTTTGTGTTTGGG/TGTTAGCTTTACTTTGATGGCC	194
CT1/PCS1	TATGTACCGCTCATCCCGA/GAAACGACGAATAACCTCTCTT	106
CT2/PCS2	TCTACGGCGGCAACTCTAT/CCACTCTCTCACAACTTTTCTCTTC	161
CT3/CYP450	TATACTCATGCGCCCAGCC/AACAGCCATCAGCAAACCT	112
CT4/HSFA4a	AACAGATGACAGAAAAGATGGG/CAGGAGCAAAGATATGATACAC	150
CT5/UGP	CCGAGGAAATGTCATAGGAGC/CAAAAACAAGTACAGACGCACC	147
*SpEF1α*	GCGGACTGTGCTGTGCTTATC/AGTGGTGGCATCCATCTTGTT	153

Under Cd-stress conditions, the expression of *PCS1* and *PCS2* in leaves decreased within 6 h and was up-regulated at 24 h. *PCS1* in roots showed significant up-regulation at 6 h, whereas the expression of *PCS2* in roots decreased at 6 h of Cd treatment and then returned to untreated levels. *CYP450* expression in leaves and roots increased at 1 and 24 h of Cd treatment, respectively. The expression of *HSFA4a* in leaves decreased at 1 h and up-regulated after 24 h, whereas *HSFA4a* expression in roots was up-regulated at 6 h. Cd-stress significantly reduced the expression of *UGP* in roots within 48 h, whereas *UGP* expression in leaves decreased at 6 h of Cd treatment and then returned to the untreated level by 24 h.

## Discussion

### Candidate genes and their biological functions associated with salinity tolerance in seashore paspalum

By screening the cDNA library of seashore paspalum using the FOX-gene hunting method in yeast, 18 salinity-tolerance related genes were identified, and most of them have not been previously reported in relation to salinity tolerance, which could be novel salinity-tolerance genes contributing to superior salinity tolerance in the halophytic grass species. These genes were found to exhibit putative functions in iron transport (*IRT* and *YS*), protein modification (*14-3-3* and *LRR*), vesicle-associated protein (*VAP33*), photosynthetic metabolism (*PSAK, LHCB*, and *Elip*), antioxidant metabolism (*CS, AKR*, and *APX*), heat shock protein (*HSP*), Nop14-like family protein (*NOP14*), choline-phosphate cytidylyltransferase (*CCT*), IQ-DOMAIN 14-likeprotein (*IQ14*), metacaspase-5-like protein (*MCP*), and BTB/POZ domain-containing protein (*BTB*). The biological functions of the aforementioned 18 genes in relation to salinity tolerance are discussed below.

#### Iron transport

Iron is an essential microelement, which is important for plant growth and development (Bashir et al., 2013). In *Arabidopsis*, iron can be assimilated through three steps: Fe^3+^ solubilization and chelation, reduction of Fe^3+^ to Fe^2+^, and Fe^2+^ uptake by divalent Fe transporter IRT1 (Brumbarova et al., 2015). In addition, in grass plants, such as rice, apart from the IRT1 pathway, Fe^3+^ can be directly absorbed by the yellow stripe-like (YSL) family of ferric iron transporters (Bashir et al., 2013). *Arabidopsis* plants lost viability in mutants with loss of function of *irt1*, which implies that IRT1 plays critical roles in iron uptake in plants (Varotto et al., 2002; Vert et al., 2002). However, both the iron transporters, IRT1 and YSL, have not been previously reported in relation to salinity tolerance of plants. A recent study with exogenous iron treatment reported that iron uptake could ameliorate salinity injury in tomato (*Solanum lycopersicum*; Ghasemi et al., 2014), whereas another study found salinity stress significantly reduced iron absorption of roots in rice (Abbas et al., 2015). In this study, *IRT1* expresion level decreased in roots of seashore paspalum under salinity stress, suggesting that salinity could interupt iron transport. Furthermore, yeast cells containing either *IRT1* or *YSL* cloned from seashore paspalum exposed to salinity stress exhibited improvement in growth in the culture medium containing NaCl. Therefore, it could be postulated that *IRT1* and *YSL* could play positive roles in salinity tolerance of seashore paspalum. The biochemical and molecular mechanisms of *IRT1*- and *YSL*-mediated salinity tolerance in plants are currently unknown, which deserve further investigation in future research.

#### Protein modification

Plant responses to salinity stress involve protein modifications in different components of phosphorylation cascades. 14-3-3 proteins are a family of conserved regulatory proteins involved in phosphorylation cascades in higher plants (Smith et al., 2011). Previous studies showed that 14-3-3 family proteins could negatively regulate freezing tolerance through repressing CBF expression (Catala et al., 2014) and salinity tolerance by reducing SOS2 kinase activity (Zhou et al., 2014), and positively regulate drought tolerance in *Arabidopsis* (He et al., 2015). In grass plants, 14-3-3-mediated abiotic stress tolerance has not been well documented. Leucine-rich repeat receptor kinases (LRR-RKs) are the largest sub-family of transmembrane receptor kinases in plants, and the regulated pathway of LRR-RKs is involved in brassinosteroids signal, wound response, and stem cell maintenance (Torii, 2004; Ogawa et al., 2008). It has recently been reported that a LRR-RKs family member from rice named Leaf Panicle 2 (LP2) could interact with aquaporin proteins and negatively influence drought tolerance (Wu et al., 2015). Overexpression of another member of the LRR-RKs family, *OsGIRL1*, led to hypersensitive responses in plants to salinity stress and heat stress (Park et al., 2014). In our study, yeast cells transformed with either a 14-3-3 gene or a LRR-RKs gene cloned from seashore paspalum subjected to salinity stress exhibited better survival than those with the empty vectors. Our results indicated that a 14-3-3 family member and a LRR-RKs member of seashore paspalum could positively regulate salinity tolerance, which is in disagreement with what was previously found in *Arabidopsis* or rice. One potential reason of the discrepancy from previous reports is that the 14-3-3 identified from seashore paspalum in our study was a novel or different member of 14-3-3 family from previously reported *Arabidopsis* and rice (Chen et al., 2006; Zhou et al., 2014). However, qPCR analysis did not detect changes in the expression level of 14-3-3 during salinity stress, thereby suggesting possible posttranslational modification regulated by salinity. The function of *14-3-3* and *LRR-RK* cloned from seashore paspalum regulating salinity tolerance deserves further confirmation.

#### Vesicle traffic pathway

Vesicle traffic is necessary for cell metabolism, growth, and development in plants and depends on a superfamily of proteins named soluble N-ethylmaleimide-sensitive fusion protein attachment protein receptors (SNAREs; Sutter et al., 2006). A previous study indicated that *AtVAM3* (belong to the Q_a_-SNARE protein family) could be induced under salinity treatment and an *atvam3* mutant displayed better tolerance to salinity stress (Hamaji et al., 2009). A recent study showed that Arabidopsis Q_*c*_-SNARE gene *AtSFT12* positively participated in salinity tolerance and Na^+^ accumulation in vacuoles (Tarte et al., 2015). In *Arabidopsis*, VAP33 family proteins (SNARE-associated elements) can interact with a sterol-binding protein ORP3a and regulate the transport of sterols from the endoplasmic reticulum to the plasma membrane (Saravanan et al., 2009). However, to our knowledge, VAP33-mediated salinity tolerance in plants has not been previously reported. In this study, a VAP33 family gene was found to be potentially involved in the improvement of salinity tolerance through its heterologous expression in yeast and in the up-regulation of this gene in both leaves and roots of seashore paspalum in response to salinity stress. However, the regulatory mechanisms of VAP33 for salinity tolerance are unknown.

#### Phospholipid biosynthesis

Phospholipids are a major component of most eukaryotic cell membranes and critical for cell functions in plants (Lin et al., 2014). Phosphatidylcholine (PC) is an important phospholipid and synthesized largely via the CDP–choline pathway, which can be converted to phosphatidic acid (PA) by phospholipase D (PLD; Craddock et al., 2015). Several previous studies indicated that PA and PLD participated in drought and salinity tolerance (McLoughlin et al., 2013; Wang et al., 2014). However, whether upstream pathways of PC regulate salinity tolerance remains unclear. In this study, a gene encoding an upstream rate-limiting enzyme CTP:Phosphorylcholine Cytidylyltransferase (CCT) for PC synthesis was identified in yeast transformed with the gene from seashore paspalum subjected to salinity stress, and its gene expression level was also significantly up-regulated under salinity stress condition. Our results suggested that *CCT* could play roles in regulating plant tolerance to salinity stress as an upstream component of phospholipid metabolism.

#### Photosynthesis

The two photosystem complexes (PS I and PS II) located in thylakoid membranes of higher plants are essential components of light reactions in photosynthesis involved in electron transport (Chen and Xue, 2012). In this study, *PSAK* (a member of the PS I complex), *LHCB* (Light-harvesting chlorophyll a/b-binding protein belonging to the photosystem II complex), and *Elip* (early light-induced protein) isolated from seashore paspalum were up-regulated in response to salinity stress. To our knowledge, *PSAK* has not been reported to be related to salinity stress. In *Arabidopsis*, LHCB are required for stomatal response to abscisic acid (Xu et al., 2012). *LHCB* gene was identified as a candidate drought-related locus through QTL analysis in pearl millet (*Pennisetum glaucum*; Sehgal et al., 2012). *Elip* is proposed to serve as protection of the photosynthetic apparatus from high light stress, and the overexpression of *MfELIP* from *Medicago sativa* subsp. facilitates improved plant tolerance to several abiotic stresses, such as freezing, chilling, osmotic stress, and high light (Zhuo et al., 2013). The up-regulation of *PSAK, LHCB*, and *Elip* by salinity stress found in this study suggested that the superior salinity tolerance of seashore paspalum could be associated with the positive regulation of proteins involved in the light reactions of photosynthesis, *PSAK, LHCB*, and *Elip*, under salinity stress.

#### Antioxidant defense and metabolism

Abiotic stresses lead to oxidative damages that can be alleviated by antioxidant enzymes, such as ascorbate peroxidase (APX; Chen et al., 2015a). *APX*-overexpression enhanced abiotic stresses tolerance of tall fescue (*Lolium arundinaceum*; Lee et al., 2007). Cysteine synthase (CS) is responsible for the final step in biosynthesis of cysteine, which produces glutathion (GSH) that also play roles in scavenging of reactive oxygen species (Noji et al., 2001). *CS-*overexpression increased plant tolerance to oxidative stress in tobacco (*Nicotiana tabacum*; Noji et al., 2001). The aldo-keto reductase (AKR) superfamily is a large enzyme group of NADP-dependent oxidoreductases with numerous roles in metabolism, and *Arabidopsis* transformed with *PpAKR1* of peach tree (*Prunus persica*) increased in salinity tolerance (Kanayama et al., 2014). The improved salinity tolerance of the salinity-sensitive yeast cells through transformation of *APX, CS*, and *ARK* from seashore paspalum suggested that antioxidant defense and metabolism regulated by those genes could play roles in the superior salinity tolerance in seashore paspalum.

#### Other pathways

Plant heat shock proteins (HSPs) serve important roles in responses to adverse environmental conditions (Timperio et al., 2008). Overexpression of a heat shock protein ThHSP18.3 from *Tamarix hispida* confers stress tolerance to salinity, drought, and heavy metals in yeast (Gao et al., 2012). Our results of HSP expression in yeast in relation to salinity tolerance are in agreement with the previous study, suggesting the postive roles of HSP in plant tolerance to salinity stress.

BTB/POZ domain-containing proteins are interaction partners with the Cullin component of the E3 ubiquitin ligase complex, and up-regulated expressions of BTB/POZ lead to up-regulation of various protease genes (Aulakh et al., 2014), whereas no reports of BTB/POZ was related to salinity tolerance. Plant metacaspases (MCPs), a family of cysteine proteases structurally related to caspases, play a positive regulatory role in biotic and abiotic stress-induced programmed cell death (Watanabe and Lam, 2011). Our data indicated that *MCP* screened by yeast might participate in salinity tolerance of seashore paspalum.

In addition, in this study, three novel unknown function genes such as Nop14-like family protein (NOP14), protein IQ-DOMAIN 14-like (IQ14), and uncharacterized protein (UP) were identified in seashore paspalum, and their biological functions and potential roles in regulating salinity tolerances deserve further analysis.

### Candidate genes for Cd tolerance identified from seashore paspalum

Five genes from Cd-tolerant clones were found with biological functions including phytochelatins synthase (PCS1, PCS2), cytochrome P450 (CYP450), a heat shock transcription factor (HSFA4a), and UDP-glucose pyrophosphorylase (UDP).

Phytochelatin synthase (PCS) is a key rate-limiting enzyme in the synthesis of phytochelatin, which can form stable complexes with heavy metals that are subsequently transported into the vacuoles (Brunetti et al., 2011). Tall fescue plants overexpressing reed (*Phragmites australis*) *PaPCS* exhibited improved tolerance to Cd (Zhao et al., 2014). Our study found two PCS family genes from seashore paspalum, indicating the involvement of PCS in Cd tolerance for the halophytic grass species.

Cytochrome P450s (CYP450) are among the largest protein coding gene families in plant genomes, catalyzing several secondary metabolism including indole alkaloids synthesis (Irmler et al., 2000) and triterpenoid saponins synthesis (Seki et al., 2015). Previous studies indicated that the accumulation of triterpene saponins of *Quillaja brasiliensis* leaves might regulate abiotic stress tolerances (de Costa et al., 2013), and triterpene saponins could regulate ROS and NO production and then induce the metallothioneins synthesis, which is related to Cd tolerance (Balestrazz et al., 2011). The heavy metal chromium induced CYP450 family gene expressions in radish (*Raphanus sativus*) roots (Xie et al., 2015). A recent study found that a cytochrome P450 member named *OsDSS1* was involved in drought stress responses in rice (Tamiru et al., 2015), but *CYP450-*mediated Cd tolerance has not been well documented. In this study, Cd-promoted *CYP450* expression in seashore paspalum suggested that CYP450 could play roles in Cd tolerance via secondary metabolism pathway such as indole alkaloids synthesis and triterpenoid saponins synthesis.

Heat shock factors are principal regulators of plant responses to several abiotic stresses (Perez-Salamo et al., 2014). In a screen for wheat (*Triticum aestivum*) genes that confer Cd tolerance to Cd-hypersensitive yeast strain identified *TaHSFA4a*, a gene which overexpression improved Cd tolerance in rice (Shim et al., 2009). It has been shown that *AtHSFA4a* of *Arabidopsis* confers enhanced tolerance to salinity and oxidative agents (Perez-Salamo et al., 2014). A *TaHSFA4a* homolog from seashore paspalum was also identified via yeast screening for Cd tolerance in this study, implying the potential roles of *HSFA4a* in Cd tolerance of seashore paspalum.

UDP-glucose pyrophosphorylase (UGPase) is a sugar-metabolizing enzyme that catalyzes a reversible reaction of UDP-glucose and pyrophosphate from glucose-1-phosphate and UTP (Payyavula et al., 2014). UGPase functions in cell wall biosynthesis and vegetative growth improvement (Li et al., 2014a,b; Payyavula et al., 2014). Although little is known of how UGPase is related to Cd tolerance, UGP gene clones from seashore paspalum subjected to Cd stress improved yeast tolerance to Cd through heterogenous expression, indicating that *UGP*-involved sugar metabolism could facilitate plant tolerance to Cd.

## Conclusions

This study identified 18 salinity-tolerance and five Cd-tolerance genes in seashore paspalum through full-length cDNA library screening in yeast and confirmation of gene expression in seashore paspalum exposed to salinity or Cd treatment using qPCR analysis. Major functional categories of the salinity-tolerance genes included iron transport, vesicle traffic, protein modification, phospholipid biosynthesis, photosynthetic metabolism, and antioxidant protection, as well as several other pathways. Cd tolerance of seashore paspalum could be related to genes for metal chelation, secondary metabolism, stress regulatory factors, and sugar metabolism. Most of those candidate genes identified in the halophytic seashore paspalum in this study are novel genes, which have not been previously reported to be related to salinity or Cd tolerance. However, the biochemical and molecular mechanisms of how these novel genes regulate salinity and Cd tolerance are unknown. Further analysis of the biological and molecular functions of those novel genes could provide further insights into survival mechanisms of halophytes to severe salinity and Cd stress, and those genes could serves as useful target genes for genetic modification of glycophytic plants for improved stress tolerance.

## Author contributions

YC, CC, ZY, and BH conceived the study and designed the experiments. YC, CC, ZT, and JL performed the experiments. YC and CC analyzed the data with suggestions by ZY, LZ, and BH. YC, CC, and BH wrote the manuscript. All authors read and approved the final manuscript.

### Conflict of interest statement

The authors declare that the research was conducted in the absence of any commercial or financial relationships that could be construed as a potential conflict of interest.
