# Supplementary material for: Functional Identification and Characterization of Genes Cloned from Halophyte Seashore Paspalum Conferring Salinity and Cadmium Tolerance
Source: Front Plant Sci. 2016 Feb 9;7:102. doi: 10.3389/fpls.2016.00102 (PMC4746305; doi:10.3389/fpls.2016.00102)
Supplement: Supplementary file 1 [file Table1.docx]

**Supplementary Material**

**Table S1:** Ions content analysis of seashore paspalum under salinity or cadmium treatment for 48 h. Lowercase letters represent significant difference (p < 0.05) under salinity stress comparing to CK, capital letters represent significant difference (p < 0.05) under cadmium stress comparing to CK.

| **Tissue** | **Elements** | | **Content (mg g^-1^ DW)** | | |
| --- | --- | --- | --- | --- | --- |
|  |  |  | **CK** | **Salinity** | **Cadmium** |
| **Roots** | | **K** | 4.236 a | 3.680 b | / |
|  |  | **Na** | 2.198 b | 15.635 a | / |
|  |  | **Ca** | 7.578 b B | 10.154 a | 8.987 A |
|  |  | **Fe** | 0.024 a A | 0.016 b | 0.011 B |
|  |  | **Cd** | 0.005 B | / | 3.254 A |
| **Leaves** | | **K** | 27.615 a | 20.152 b | / |
|  |  | **Na** | 1.274 b | 11.375 a | / |
|  |  | **Ca** | 4.465 b A | 5.662 a | 4.724 A |
|  |  | **Fe** | 0.085 a A | 0.079 a | 0.054 B |
|  |  | **Cd** | 0.003 B | / | 1.978 A |
